# Proarrhythmogenic Echocardiographic Markers in Metabolic Syndrome: A Cross-Sectional Study

**DOI:** 10.3390/life15091443

**Published:** 2025-09-15

**Authors:** Spas Kitov, Maria-Florance Kitova, Boyan Nonchev, Mariya Tokmakova, Lyudmila Kitova

**Affiliations:** 1 Cardiology Clinic, St. George University Hospital, Faculty of Medicine, Medical University of Plovdiv, 4002 Plovdiv, Bulgaria; kitovspas@yahoo.com (S.K.); m14021971@yahoo.co.uk (M.T.); 2 Clinic of Cardiology, St. George University Hospital, 4002 Plovdiv, Bulgaria; 3 Faculty of Medicine, Medical University of Plovdiv, 4002 Plovdiv, Bulgaria; maria_kitova21@abv.bg; 4 Clinic of Endocrinology and Metabolic Diseases, “Kaspela” University Hospital, Medical University of Plovdiv, 4001 Plovdiv, Bulgaria; nonchev_md@abv.bg

**Keywords:** keyword metabolic syndrome, proarrhythmogenicity, mechanical dispersion index, global longitudinal strain, left atrial reservoir strain, left atrial conduit strain, left atrial contractile strain

## Abstract

In metabolic syndrome, cardiomyocyte changes induced by metabolic and proinflammatory factors impair repolarization and exacerbate the heterogeneity of the transmural dispersion of repolarization, and this is proarrhythmogenic. Limited data in the literature on the capabilities of speckle tracking echocardiography for assessing proarrhythmogenicity in metabolic syndrome exists. 71 patients with newly diagnosed metabolic syndrome, aged 35–55 years, were studied. Ischemic heart disease was excluded in all patients with stress test cycle ergometry, CT-angiography or selective coronary angiography. All patients underwent a 48-h Holter ECG recording. Based on the latter, they were divided into two groups: 38 patients (53.5%) with a high arrhythmogenic load (supraventricular or ventricular tachycardia, atrial fibrillation/flutter, ventricular extrasystoles over 10%, frequent supraventricular extrasystoles > 500/24 h are included); and 33 patients (46.5%) with low arrhythmogenic load (no significant rhythm disturbances are included). Echocardiography was performed with a GE Vivid T9 emphasizing global longitudinal strain, mechanical dispersion index and left atrium strains. Statistically significant differences in the global longitudinal strain, mechanical dispersion index, and left atrium strain were found between the group with low arrhythmogenicity and the group with high arrhythmogenicity (*p* < 0.0001). The index of mechanical dispersion has the most optimal sensitivity and specificity of all investigated echocardiographic markers. These results support the mechanical dispersion index as an additional tool for assessing proarrhythmogenicity in metabolic syndrome.

## 1. Introduction

Metabolic syndrome (MetS) is a socially significant condition, with its prevalence steadily rising worldwide. According to the World Health Organization, the prevalence of metabolic syndrome in Europe according to the criteria of the International Diabetes Federation is 31.5% [1,2]. It represents a cluster of cardiovascular risk factors—abdominal obesity, hyperglycemia, hypertension, and hypertriglyceridemia. The presence of three or more of these components is associated with elevated circulating proinflammatory cytokine levels [1,2].

MetS extends beyond metabolic disturbances alone; it involves multiple pathophysiological mechanisms that influence cardiac electrophysiology and contribute to proarrhythmogenicity [3]. A key factor is adipocyte dysfunction, which promotes inflammatory activity. Metabolic and proinflammatory influences on cardiomyocytes disrupt repolarization, increase transmural repolarization heterogeneity, and thereby create a proarrhythmic substrate [3].

Echocardiography—being non-invasive, widely accessible, and reproducible—remains an essential tool for evaluating structural arrhythmogenic risk, particularly through ejection fraction, but only when EF falls below 35% [4,5,6,7,8,9,10,11]. However, in patients with preserved EF, sudden cardiac death still occurs in 30–40% of cases. This underscores the need for more sensitive echocardiographic parameters to guide arrhythmia risk stratification. Several novel echocardiographic markers are currently being explored for this purpose [9,10,11].

Speckle tracking echocardiography offers important advantages for evaluating myocardial function and arrhythmogenic risk. Global longitudinal strain (GLS) has been recognized for more than 15 years as a reliable parameter for assessing global left ventricular performance and detecting subclinical dysfunction [12,13,14,15]. Despite variations in study endpoints, monitoring methods, and follow-up durations, GLS has consistently been shown to predict ventricular arrhythmias [8,10,16,17,18,19,20]. However, data on GLS in the context of metabolic syndrome are limited, and evidence specifically addressing its proarrhythmogenic potential in this condition is even scarcer [21,22].

In addition to GLS, mechanical dispersion index (MDI) is frequently evaluated. MDI is defined as the standard deviation of the time to peak longitudinal strain and is closely linked to myocardial fibrosis, which in turn increases the risk of ventricular arrhythmias. Fibrosis causes electrical dispersion and heterogeneous contraction patterns, contributing to arrhythmogenicity [23]. Although reference data for MDI in healthy individuals remain limited, it has been primarily investigated as a predictor of ventricular arrhythmias [24]. Like GLS, the association between MDI and arrhythmic risk has been well established across various populations, including patients with heart failure [15,25], ischemic heart disease [26], long QT syndrome [27], arrhythmogenic cardiomyopathy [28], and congenital heart disease [29].

On the other hand, obese patients have impaired left atrial function before changes in conventional echocardiographic parameters occur [30,31,32,33,34,35,36,37,38,39,40,41,42,43]. Atrial fibrillation is the most common arrhythmia, and one of its manifestations is increased left atrial filling pressure and left atrial dilatation.

There is ample evidence in the literature that echocardiography is not only a method for assessing the structural and functional characteristics of the heart, but also for the proarrhythmogenic load. This echocardiographic assessment is very important for assessing the risk of rhythm disorders and sudden cardiac death, but it is still a great challenge. Furthermore, obesity is the new pandemic and as a major part of the metabolic syndrome, the assessment of proarrhythmogenicity in daily clinical practice is significant. In the primary prevention setting, this is extremely valuable for changing the prognosis of patients with metabolic syndrome. There is sufficient evidence that speckle tracking echocardiographic techniques have informative value for the proarrhythmogenic load, but mainly in secondary prevention setting [10,11,16,17,18,19].

There is limited data in the literature about the proarrhythmogenic potential on GLS, MDI and left atrial strain in patients with metabolic syndrome [29,30]. The latter is likely related to the difficult echocardiographic imaging in these patients. The present study aims to shed light on the significance of these new echocardiographic parameters (GLS, MDI and left atrial strain) in assessing proarrhythmogenicity in newly diagnosed asymptomatic metabolic syndrome.

## 2. Materials and Methods

**Figure 1:**
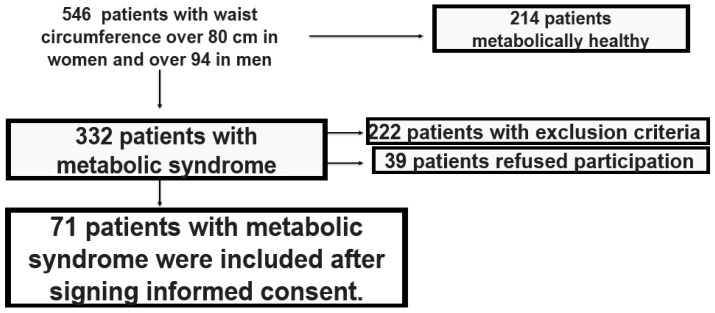
Patient screening algorithm.

We gathered data from outpatients of the Clinic of Cardiology at St. George University Hospital in Plovdiv, Bulgaria. Patients who met the criteria of the International IDF for MetS were recruited between September 2023 and September 2024 [2]. In order to mitigate confounding effects, we excluded individuals with chronic renal, hepatic, respiratory, cardiac, thyroid diseases, cancer, pregnancy, electrolyte imbalance, recent infections, and those taking medications known to affect electrocardiographic repolarization. Of the 546 obese individuals who were screened, 214 were excluded on the basis that they did not have MetS, 222 met some of the exclusion criteria, and 39 refused to participate in the study. In total, 71 participants were eligible and were used for the present secondary analysis. Participants underwent comprehensive clinical, anthropometric, and laboratory examinations. The patient screening algorithm is presented in Figure 1.

Seventy-one patients with asymptomatic metabolic syndrome aged 35–55 years were studied. The absence of coronary heart disease as an inclusion criterium was added as it allows for better correlation between obesity and proarrhythmogenicity in newly diagnosed metabolic syndrome. The lack of coronary obstructions and myocardial ischemia eliminates ischemia as a cause for arrhythmias in these patients. The exclusion criteria eliminate all other causes associated with the development of arrhythmias. Ischemic heart disease was excluded in all patients by stress test cyclic ergometry, CT angiography or selective coronary angiography. Invasive coronary artery diagnostics were performed in only one patient due to a highly suspicious stress test. All patients underwent 48-h Holter ECG recording. Based on the latter, they were divided into two groups-with high arrhythmogenic load-38 patients (53.5%) (includes supraventricular or ventricular tachycardia, atrial fibrillation/flutter, ventricular extrasystoles over 10%, frequent supraventricular extrasystoles over 500/24 h) and with low arrhythmogenic load-33 patients-46.5% (includes no significant rhythm disturbances).

### 2.1. Ethical Approval

The study was reviewed by the Scientific Ethics Committee at the Medical University of Plovdiv and received a positive statement (Protocol No. 9/28, 13 November 2023).

### 2.2. Laboratory Diagnostic Tests

Blood counts (hemoglobin, erythrocytes, leukocytes, platelets), insulin resistance markers (blood sugar, immunoreactive insulin, HOMA index, glycated hemoglobin, total cholesterol, triglycerides (TG), HDL-cholesterol, LDL-cholesterol, non-HDL-cholesterol, apolipoprotein-A1, apolipoprotein-B), thyroid hormones (TSH, FT3, FT4), which served to fulfill the exclusion criteria and determine the severity of METS, were examined. The studies were conducted in the Central Institute Clinical Laboratory at St. George University Hospital-Plovdiv. Accordingly, hematological analyses (leukocytes, erythrocytes, platelets; determination of hemoglobin concentration, haematocrit) were performed on an automatic hematological analyser Advia 2120 (Siemens Healthcare, Erlangen, Germany). Serum biochemical parameters were examined on a clinical chemistry analyser AU 480, Beckman Coulter (Brea, CA, USA) using original prograMetS with conventional analytical principles of the methods used. Hormonal studies: principle of the analyses: competitive immunochemical analysis of the chemiluminescent principle (CLIA) on an immunological analyser Access 2, Beckman Coulter, Brea, CA, USA.

### 2.3. Inflammatory Mediators and Adipokines

Proinflammatory cytokines (IL-6, TNF-α), high-sensitivity CRP, and adipokines (visfatin, leptin, and adiponectin) measured at the Central Clinic Laboratory of the St. George University Hospital were studied. Venous blood samples were collected in accordance with standard procedures and serum from each participant was aliquoted and stored at −20 °C until analysis, with a maximum storage time of two months. Serum CRP levels were measured by immunoturbidimetry using an AU 480 Beckman Coulter clinical chemistry analyser. The serum concentrations of TNF-α, IL-6, leptin (LDN, Nordhorn, Germany), visfatin, and high sensitivity adiponectin were determined using competitive enzyme-linked immunosorbent assay (BioVendor, Asheville, NC, USA), following validation at the local level. We also calculated the adiponectin-leptin ratio as a marker of dysfunctional adipose tissue [44,45].

### 2.4. Adiposity-Related Markers

Anthropometric measures included body mass index (BMI), waist-to-hip ratio (WHR), total body fat, and visceral fat. These measurements were taken at the Department of Physiology of the Medical University of Plovdiv. BMI (i.e., weight in kilograMetS divided by height in meters squared) was calculated from weight and height measured barefoot and in light clothing. WHR was calculated from waist circumference measured at the midway between the last palpable rib and the iliac crest and hip circumference [46].

Body fat percent (BF%) and visceral fat percent (VF%) were estimated based on multifrequency bioelectrical impedance analysis using an InBody_270_ analyser (InBody Co, Ltd., Seoul, Republic of Korea). This technique uses varying frequencies of alternating current that are sent through the body, where passing through different tissues alters the resulting voltage, which information is later used to predict BF% and VF%. This renders reasonable estimates compared with the reference method dual-energy X-Ray absorptiometry [47].

Echocardiography was performed with a Vivid E9 echocardiograph (Chicago, IL, USA, GE Ultrasound). To optimize image quality in METS, the following important conditions were considered:

1. Minimize translational motion of the heart by using lower frequency transducers (below 2.5 MHz); adjust gain, dynamic range, transmission, and side gain controls appropriately; frame rate ≥ 30/s; harmonic imaging
2. Maximize endocardial border delineation-tissue harmonic imaging and contrast echocardiography
3. Identify end diastole and end systole-using ventricular cavity size and mitral valve motion, not ECG (end diastole = maximum size and end systole = minimum size)

Echocardiography included conventional transthoracic echocardiography at rest with assessment of cavity sizes/diameters of the left ventricle, left atrial area, right ventricle diameter, right atrial area, function-ejection fraction by the Teichholz and Simpson method, MAPSE, TAPSE, diastolic function assessment parameters-E/A, E/e′. With 2D echocardiography, speckle-tracking Global Longitudinal Strain was assessed. To assess longitudinal strain, three standard images (apical two-, three-, and four-chamber) recorded during three consecutive beats were analyzed and the analysis was performed using the Automatic Functional Image Model (AFI). In this method, the left ventricular contour was traced in each view and after some manual endocardial adjustment, the software automatically provided a result for each myocardial segment [9]. Finally, the peak values of systolic longitudinal strain for each segment were recorded in the form of a 17-segment bull’s-eye, which was designated as segmental longitudinal strain. The global longitudinal strain was automatically calculated as the average of three apical projections. A value ≥ 20% for the global longitudinal strain was taken as the reference. Only patients with normal global longitudinal strain, i.e., ≥20%, were included in the study. Left ventricular mechanical dispersion was also measured in parallel with global longitudinal strain [9]. The reference value for the left ventricular mechanical dispersion index in patients with normal EF and normal GLS is up to 85 ms [9].

Left atrial strain was measured with a Vivid E9 echocardiograph (Chicago, IL, USA, GE Ultrasound). An apical 4-chamber image and 2-chamber image were used, and the average value was taken accordingly. The endocardial borders of the left atrium were automatically tracked using end-diastole as a reference value. When tracking was suboptimal, fine-tuning was performed manually. Patients with images of insufficient quality for performing left atrial strain analysis were excluded.

Left atrial function was described according to the three phases of the left atrial cycle: left atrial reservoir strain (LASr), which begins at the end of ventricular diastole (mitral valve closure) and continues until mitral valve opening, left atrial conduit strain (LAScd), which occurs from the moment of mitral valve opening through diastasis to the beginning of left atrial contraction, and left atrial contractile strain (LASct), which occurs from the beginning of left atrial contraction to the end of ventricular diastole (mitral valve closure). LASr, LAScd, and LASct were calculated in all patients. Left atrial dysfunction was defined as LASct < 14% [48].

### 2.5. Statistical Methods

We applied descriptive statistical methods to present summary characteristics for quantitative (metered) indicators, with the results presented as arithmetic mean ± standard error (mean ± SEM). Some characteristics of the sample distribution are also presented, such as the number of studied units (n), minimum value (Minimum), maximum value (Maximum) and the values between the 25th and 75th percentile (IQR).

Descriptive statistical methods for qualitative (non-metered) indicators were applied, presenting the summary characteristics through absolute frequencies and relative shares. Student’s t-test (independent samples t-test) was applied to assess a statistically significant difference between the groups with low and high arrhythmogenicity for indicators with a normal distribution. ROC analysis was applied to assess the predictive power of the studied indicators, presenting the ROC curve and evaluating the coordinates of the points on it. The area under the curve for each predictor analyzed is also presented. To check the distribution normality of the analyzed quantitative indicators, we applied the Kolmogorov–Smirnov test. The Mann–Whitney test was applied for comparison of quantitative values in independent samples with a distribution different from normal.

The significance level of the null hypothesis was *p* < 0.05. All statistical analyses were performed using IBM SPSS Statistics (Version 28).

## 3. Results

Table 1 presents the baseline demographic and anthropometric data, concomitant diseases, lipid and diabetes profile, inflammatory markers, echocardiographic parameters of the two groups with low and high arrhythmogenicity. The patients did not receive concomitant therapy because they were recently diagnosed with metabolic syndrome.

When comparing the two groups with high and low arrhythmogenicity by gender, age, anthropometric indicators, number of components of the metabolic syndrome, family history, smoking, metabolic biomarkers (blood sugar (Gl), immunoreactive insulin, HOMA index, glycated hemoglobin, total cholesterol, triglycerides (TG), HDL-cholesterol, LDL-cholesterol, non-HDL-cholesterol, apolipoprotein-A1, apolipoprotein-B) and atherogenic indices (TG/HDL, TC/HDL, LDL/HDL, LDL/Apo-B, TG/Gl, no statistically significant difference was found. (*p* > 0.05)

**Figure 2:**
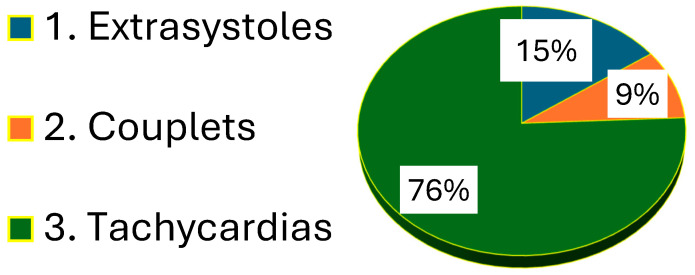
Distribution of rhythm disorders in the group with high arrhythmogenicity.

The following distribution of rhythm disturbances was found in the group with high arrhythmogenicity (Figure 2).

The two groups with different arrhythmogenicity were compared by the basic echocardiographic indicators used in daily clinical practice–left ventricular end-diastolic diameter, E/A, E/e′, septal velocity e′, tricuspid blood flow velocity, left atrial volume, left atrial volume indexed compared to height (h^2^), no statistically significant difference between the groups with low and high arrhythmogenicity was found. (*p* > 0.05) (Table 2).

For the remaining studied echocardiographic parameters–GLS, MDI, left atrial strain–reservoir, contractile and conduit, statistically significant differences were found between the two groups with different arrhythmogenicity. The analysis of the distribution of the listed studied echocardiographic parameters (Kolmogorov–Smirnov test) shows that the distribution differs statistically from the normal distribution, which is why a parametric analysis (Mann–Whitney test) was applied in the comparison. Therefore, data for Median and Interquartile Range are additionally presented in Table 3. For all analyzed parameters, a statistically significant difference was found between the groups with low and high arrhythmogenicity.

From the studied echocardiographic parameters, a statistically significant difference was found in the three types of left atrial strain–reservoir, conduit and contractile. In the group with high arrhythmogenicity, statistically lower values of all three parameters were observed (*p* < 0.0001).

A multivariate logistic regression analysis was performed to exclude confounding factors.

**Figure 3:**
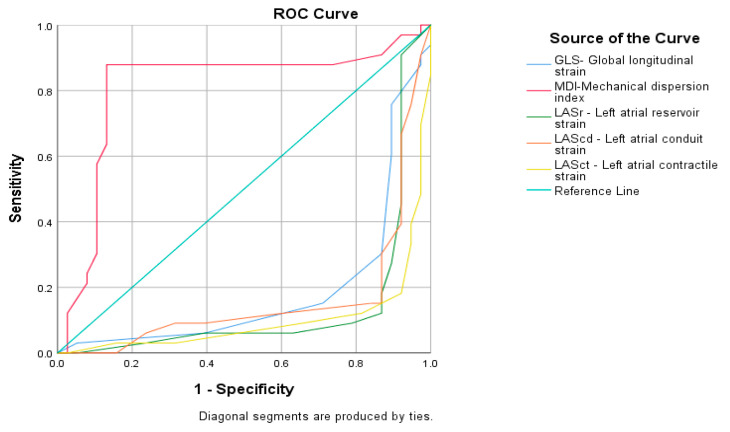
ROC curves of global longitudinal strain (GLS), mechanical dispersion index (MDI), LA_r- left atrial reservoir strain (LASr), left atrial conduit strain (LAScd), and left atrial contractile strain (LASct), MDI demonstrated a very large AUC (AUC=0.808 sec; CI 95 %, 0.691–0.925; *p* < 0.0001).

The results of the ROC curve, including all five echocardiographic parameters, only for MDI demonstrated a very large AUC (AUC=0.808 sec; CI 95 %, 0.691–0.925) and high statistical significance (*p* < 0.0001) (Figure 3).

**Figure 4:**
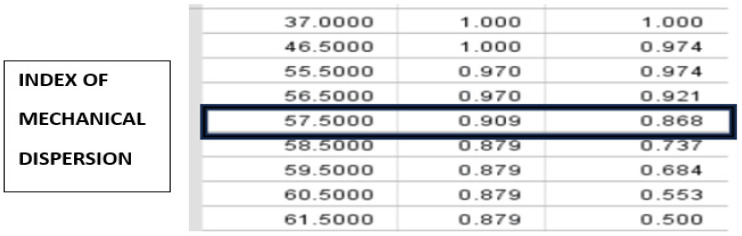
Cut-off 57.50 ms for mechanical dispersion index at optimal sensitivity (0.909) and specificity (0.868).

When choosing the optimal sensitivity and specificity for MDI interval, a duration of 57.50 ms is established as the limit above which arrhythmogenicity increases in MetS patients (Figure 4).

## 4. Discussion

In the present study, patients with asymptomatic metabolic syndrome showed statistically significant differences across all parameters measured by speckle tracking echocardiography—GLS, MDI, and the three types of left atrial strain (reservoir, conduit, and contractile) (*p* < 0.0001). Even when limited to newly diagnosed asymptomatic cases, GLS values differed statistically significantly between the two groups (*p* < 0.0001).

Currently, there is limited evidence in the literature regarding the proarrhythmogenic potential of GLS in metabolic syndrome, which this study discusses. Among all the echocardiographic measures analyzed (such as left ventricular end-diastolic diameter, E/A, E/e′, septal velocity e′, maximum tricuspid regurgitation velocity, and left atrial volume indexed to height^2^; (Table 2), GLS was the first parameter to show a statistically significant difference between patients classified as low- and high-risk for arrhythmias, despite both groups having normal EF and GLS. This finding is consistent with the role of GLS in evaluating longitudinal deformation, which is closely linked to arrhythmogenic potential [49].

Previous research also indicates that metabolic syndrome is independently associated with GLS, with visceral obesity being the strongest factor connecting metabolic abnormalities to impaired cardiac function [21]. For example, Shen et al. reported that metabolic syndrome correlates with reduced GLS in patients with non-ischemic dilated cardiomyopathy [21].

Regarding MDI, the present study demonstrates a statistically significant difference between the low and high proarrhythmogenic groups in asymptomatic newly diagnosed metabolic syndrome. The data demonstrate more prolonged MDI values in the high proarrhythmogenic group (79.82 ± 1.83 MetS) compared to the low proarrhythmogenic group (64.00 ± 1.72 ms) (*p* < 0.0001). The MDI levels in both groups were lower than the recommended upper limit in the literature of 85 ms for normal GLS [9]. This can be explained by the characteristics of the studied population of patients with metabolic syndrome, in which the ROC curve gives 57.50 ms above which arrhythmogenicity increases in patients with MetS. Based on this data, it can be assumed that in MetS the limit above which arrhythmogenicity increases is much lower than in the general population [47]. In newly diagnosed asymptomatic metabolic syndrome, this result is probably related to the initial fibrosis of the left ventricle, which can lead to a heterogeneous contraction pattern, since fibrosis leads to electrical dispersion, affecting both activation time and refractoriness.

The results obtained in the present study for the arrhythmogenicity limit in newly diagnosed asymptomatic metabolic syndrome are close to some data in the literature for the upper reference limit in healthy individuals. Studies examining normal reference values based on healthy individuals are scarce. A study by Rodrígues-Zenella et al. sought to define reference values. Based on the data from 334 healthy volunteers (Caucasian, mean age 54 (range: 18–79) years, and 54% female), the authors found an overall normal value of 34 ± 10 msec with an upper limit of 56 ms [24].

The present study demonstrates this association, albeit in a small number of patients (*n* = 71). Moreover, of the echocardiographic parameters studied, MDI is the only one that, with high sensitivity and specificity above a certain value of 57.50 ms, can predict proarrhythmogenicity. The developing obesity cardiomyopathy in asymptomatic newly diagnosed metabolic syndrome probably occurs very early and the resulting fibrosis reflects a heterogeneous pattern of contraction, as fibrosis leads to electrical dispersion, affecting both activation time and refractoriness. The ability to diagnose these early changes in metabolic syndrome provides an opportunity to prevent future arrhythmias and reduce the risk of sudden cardiac death [50,51,52,53].

There is scant data in the literature on whether MDI is a good predictor of ventricular arrhythmias in ischemic and non-ischemic cardiomyopathy. On the one hand, in ischemic cardiomyopathy, Kawakami et al., in a review and meta-analysis which included 12 studies with 3198 patients and 387 arrhythmic events over 17 to 70 months of follow-up, found that MDI was higher in patients with ventricular arrhythmias, that MDI was independently associated with ventricular arrhythmias, and that MDI was superior to the left ventricle EF and GLS for this purpose [30]. On the other hand, in a meta-analysis, Harapoz et al. reported the potential of MDI in patients with non-ischemic cardiomyopathy (n: 346 with 107 events), in which no significant association between MDI and ventricular arrhythmias was found [54]. This raises the question of whether MDI is as good a predictor of ventricular arrhythmias in non-ischemic cardiomyopathy as in ischemic cardiomyopathy. The two types of cardiomyopathies differ in terms of the underlying pathophysiology and the distribution of fibrosis. There is limited data on the association between MDI and arrhythmogenicity in metabolic syndrome.

According to literature data, not only baseline MDI examination is important, but also the timing of the examination, i.e., its monitoring. The current study lacks patient follow-up. Kutyifa et al. reported that baseline MDI is not predictive of ventricular arrhythmias but noted that patients with CRT with left bundle branch block who had improvement in MDI at 12 months had a lower risk of ventricular arrhythmias [55]. Similar findings were reported in a large retrospective study by Van der Bijl et al. Based on 1185 patients with 403 events, they did not find that baseline MDI was associated with ventricular arrhythmias, but rather that MDI at 6 months was independently associated with ventricular arrhythmias [26]. Accordingly, the timing of echocardiography appears to be important when considering the use of MDI to predict ventricular arrhythmias.

A statistically significant difference was found in the three types of left atrial strain—reservoir, conduit, and contractile- of all the echocardiographic parameters studied. The high arrhythmogenic group had statistically lower values for all three parameters. Our results are similar to those described by Hyun-Jin Kim & Hyun-Sun Kim [56]. The reduction in left atrial strain can be interpreted as a discrete mechanical dysfunction at the cellular level due to compromised intracellular ion homeostasis.

Plasek et al. showed that left atrial longitudinal strain was able to differentiate patients with paroxysmal atrial fibrillation from patients without a history of this arrhythmia [44]. In recent years, there has been a significant increase in the literature on left atrial strain and atrial fibrillation, the most common arrhythmia. The relationship between elevated levels of inflammatory and metabolic markers in MetS and the structurally more vulnerable left atrium than the left ventricle is logical. There is little data in the literature on the relationship between arrhythmogenicity and left atrial strain in patients with metabolic syndrome.

The results of the present study showed a smaller decrease in left atrial reservoir strain in patients with low arrhythmogenicity (36.32 ± 0.93) and a greater decrease in patients with high arrhythmogenicity (25.39 ± 0.91). Our data confirms the data of Aga et al. (2023) in patients without cardiovascular disease, that obese patients have a significant decrease in left atrial strain parameters compared to non-obese controls [31]. The authors found that left atrial reservoir strain was significantly lower compared to controls (32.2 ± 8.8% vs. 39.6 ± 10.8% *p* < 0.001). The LASr values found in the present study are similar to those of Aga et al., but in the latter, there is no separation according to arrhythmogenicity. This suggests that functional changes in the left atrium may occur before the development of cardiovascular disease. The results of our study add additional information about the arrhythmogenic potential of left atrial strain to the data of Aga et al. However, when constructing the ROC curve in our patients, neither of the left atrial strains showed a relationship to prognosis.

The current study also has a number of limitations. First, it was conducted with a small number of patients and at only one center. Second, briefly acknowledge the lack of longitudinal follow-up and potential selection bias due to exclusion criteria.

There are two clinical messages from this article:

1. Speckle tracking echocardiography in metabolic syndrome can be used as proarrhythmogenic screening in newly diagnosed asymptomatic metabolic syndrome.
2. Mechanical dispersion index with duration over 57.50 msec may be useful in the clinical prognostic assessment of proarrhythmogenicity in newly diagnosed metabolic syndrome. Their determination during routine echocardiography should not be underestimated.

The data from this study confirm the fact that by assessing left ventricular deformation we obtain information not only about morphology and function, but also about proarrhythmogenic potential. Our data also proves this in patients with newly diagnosed metabolic syndrome, for which there is very limited data in the literature. Additional larger studies are needed to confirm this data and to establish the limits above which the risk of arrhythmias increases in metabolic syndrome.

## 5. Conclusions

The mechanical dispersion index assessed via speckle tracking echocardiography may be a valuable noninvasive tool for assessing proarrhythmogenic risk in newly diagnosed asymptomatic metabolic syndrome. These claims need to be confirmed in larger validation studies.

## Figures and Tables

**Table 1:** Baseline demographic and anthropometric data, concomitant diseases, lipid and diabetes profile, inflammatory markers, echocardiographic parameters of the two groups with low and high arrhythmogenicity.

	Low Arrhythmogenicity (n = 38)	High Arrhythmogenicity (n = 33)
Male/Female, n (%)	20/18 (52.6%/47.4%)	23/10 (69.7%/30.3%)
Age, year	45.47 ± 1.11	45.97 ± 1.32
BMI (kg/m^2^)	38.71 ± 1.54	40.85 ± 1.22
Waist circumference (sm)	108.10 ± 19.52	129.43 ± 20.35
Waist-hip ratio	1.07 ± 0.015	1.09 ± 0.012
Fat percentage (%)	39.26 ± 1.32	38.92 ± 1.31
Internal fat level	16.89 ± 0.57	18.15 ± 0.50
Arterial hypertension (%)	34.83± 31.47	28.25 ± 29.08
Diabetes Mellitus (%)	35.12± 25.33	33.99 ± 29.55
Total cholesterol (mmol/L)	5.76 ± 1.34	5.8 ± 1.09
Triglycerides (mmol/L)	2.75 ± 1.9	2.0 ± 0.95
HDL-cholesterol (mmol/L)	1.2 ± 0.24	1.27 ± 0.27
LDL-cholesterol (mmol/L)	3.38 ± 1.23	3.7 ± 0.86
Non-HDL-cholesterol	4.15 ± 1.23	4.55 ± 0.86
Apolipoprotein-A_1_ (g/L)	1.54 ± 0.40	1.52 ± 0.34
Apolipoprotein-B (g/L)	1.33 ± 0.39	1.32 ± 0.53
Apolipoprotein- B/A_1_ ratio	0.92 ± 0.38	0.97 ± 0.72
TG/HDL ratio	2.47 ± 2.03	1.68 ± 1.02
TG/GL ratio	0.47 ± 0.34	0.35 ± 0.16
LDL/HDL ratio	2.843 ± 0.15	3.06 ± 0.16
LDL/ApoB ratio	2.7± 0.17	3.21 ± 0.21
TG/HDL ratio	2.47 ± 2.03	1.6 ± 1.02
Glucose (mmol/L)	5.9 ± 1.47	5.7 ± 1.34
НОМА index	5.17 ± 4.27	6,5 ± 7.3
Glycated hemoglobin (%)	5.6 ± 0.76	5.7 ± 0.66
Immunoreactive insulin (μU/mL)	18.66 ± 12.47	23.81 ± 19.55
Adiponectin (µg/mL)	8.1 ± 3.8	7.9 ± 3.31
Leptin (ng/mL)	64.7 ± 51.12	74.8 ± 57.9
Adiponectin/Leptin	1.7 ± 1.42	0.20 ± 0.04
hsCRP (mg/dL)	9.8 ± 3.3	8.9 ± 4.0
Visfatin (ng/mL)	5.97 ± 3	5.62 ± 2.7
Interleukin-6 (pg/mL)	15.9 ± 10.16	58.08 ± 77.75
TNF- α (pg/mL)	6.5 ± 7.08	26.96 ± 30.59
Left ventricular endodiastolic size (sm)	4.867 (0.148–0.179)	4.217 (0.136–0.227)
E/A	0.68 (0.56–0.89)	0.64 (0.50–0.87)
E/e‘	17.5 (13.75–18)	18.7 (15–19)
Septal velocity e‘ (m/s)	6.5 (5.3–7.25)	4.5 (4.9–7.5)
V max tricuspid insufficiency (m/s)	2.0 (1.75–2.5)	2.05(1.6–2.6)
Left atrial volume indexed for height^2^	19.5 (17.6–22.5)	21.7 (18.5–22.5)
GLS (%)	21.9 ± 1.3	20.1 ± 1.3
MDI (ms)	64 ± 10.5	79.8 ± 10.5
LASr (%)	36.31 ± 5.7	25.3 ± 5.2
LAScd (%)	20.3 ± 3.4	14.5 ± 3.7
LAct (%)	15.7 ± 1.9	11.2 ± 2.6

**Table 2:** Baseline echocardiographic parameters in the two studied groups.

**Group**	**Low Arrhythmogenicity** **(n = 38)**	**High Arrhythmogenicity** **(n = 33)**	***p*-Value**
Echo-marker	Median (25th–75th)	Median (25th–75th)	
Left ventricular endodiastolic size (sm)	4.867 (0.148–0.179)	4.217 (0.136–0.227)	0.44
E/A	0.68 (0.56–0.89)	0.64 (0.50–0.87)	0.854
E/e′	17.5 (13.75–18)	18.7 (15–19)	0.045
Septal velocity e′	6.5 (5.3–7.25)	4.5 (4.9–7.5)	0.55
V max tricuspid insufficiency	2.0 (1.75–2.5)	2.05 (1.6–2.6)	0.712
Left atrial volume indexed for h^2^	19.5 (17.6–22.5)	21.7 (18.5–22.5)	0.913

**Table 3:** Comparison of echocardiographic parameters between the group with low and high arrhythmogenicity.

**Group**	**Low Arrhythmogenicity** **(*n* = 38)**	**High Arrhythmogenicity** **(*n* = 33)**	***p*** **-Value**
Marker	mean ± SEM	mean ± SEM	
GLS	21.90±0.23	20.17 ± 0.23	<0.0001
MDI	64 ± 1.72	79 ± 1.83	<0.0001
LASr	36.32 ± 0.93	25.39 ± 0.91	<0.0001
LAScd	20.38 ± 0.56	14.52 ± 0.66	<0.0001
LASct	15.74 ± 0.31	11.21 ± 0.46	<0.0001

## Data Availability

The original contributions presented in this study are included in the article. Further inquiries can be directed to the corresponding author.

## Abbreviations

The following abbreviations are used in this manuscript:

MetS	Metabolic syndrome
GLS	Global longitudinal strain
MDI	Mechanical dispersion index
LASr	Left atrial reservoir strain
LAScd	Left atrial conduit strain
LASct	Left atrial contractile strain
